# Barriers to knowledge translation: from controlled scenario to clinical scenario in addictions treatment

**DOI:** 10.1186/1940-0640-10-S2-O41

**Published:** 2015-09-24

**Authors:** Kalina Isela Martínez Martínez, Aymé Pacheco Trejo, María Elena Medina-Mora Icaza, Francisco Javier Pedeoza Cabrera

**Affiliations:** 1 Psychology, Universidad Autónoma de Aguascalientes, Aguascalientes, México; 2 Psychology, Universidad Modelo, Merida, Yucatán, México; 3 Instituto Nacional de Psiquiatría Ramón de la Fuente Muñiz, México, D.F., México

**Keywords:** Brief intervention, technology transfer, addictions, barriers, KT

## Background

The transfer and translation of programs with scientific evidence to clinical scenarios is often limited by a number of barriers to their implementation [1], making it difficult to provide benefits for a society that requires effective services [2]. The aim of this study was therefore to identify barriers to the adoption of brief intervention programs for the treatment of the abuse of alcohol and other drugs [3,4] at addiction treatment centers in Mexico. On the basis of the experience of experts in the implementation and dissemination of programs, a number of actions are established to address them and advance the transfer process.

It is a qualitative study using interviews. The results reported several barriers to the adoption of programs in clinical scenarios.

The study discusses the fact that the technology transfer process requires a deliberate, combined effort to ensure the implementation of programs in clinical scenarios. The barriers identified by the actors involved in the process should be considered in the development of strategies to disseminate brief intervention programs.

## Material and methods

The team used an interview guide that included: 1. Knowledge about brief intervention programs, 2. Knowledge about working with brief intervention programs based on scientific evidence, 3. Training and supervision got during the transfer process about brief interventions, 4. Modifications to programs, 5. Barriers on the implementation of programs, 6. Advantages and limitations of brief intervention programs, 7. Other important information.

Sixteen interviews were conducted and recorded for about two hours each in the working place of the participants (therapists), who were informed about the recording, the confidentiality and the use pretended for the information. The interviews were transcribed, read, analysed, organized in categories.

## Results

The participants reported three types of barriers to the brief programs operation: Institutional barriers, Therapist barriers, Barriers in the researcher himself and barriers in the program users.

Regarding the Institutional barriers therapists report bureaucracy, lack of budget for material or for adequate working areas.

About the therapist barriers the therapists report that they own few information about the cognitive-behavioral model; they don't have affinity with the model, lack of time in the capacitation and lack of feedback.

Finally therapist reported the next researcher barriers: differences between the researcher and population goals.

## Conclusions

Findings show the point of view of the people often forgotten in the process of knowledge transfer. It is important to remark also the necessity that the therapist and the organizations be aware of the new knowledge. Also about The impact of the implementation of new technology in the efficacy of any treatment.

In the other hand, the researcher needs to take into account that the therapist has an practical vision of the phenomenon.

The barriers found in this paper, can be beaten by creating longitudinal capacitation strategies like manuals, and theoretical meetings. It is also needed a bigger budget for more therapists, material, and better work buildings.
